# Establishment of the Variation of Vitamin K Status According to *Vkorc1* Point Mutations Using Rat Models

**DOI:** 10.3390/nu11092076

**Published:** 2019-09-03

**Authors:** Jean Valéry Debaux, Abdessalem Hammed, Brigitte Barbier, Thomas Chetot, Etienne Benoit, Sébastien Lefebvre, Virginie Lattard

**Affiliations:** 1 USC 1233 RS2GP, INRA, VetAgro Sup, University of Lyon, F-69280 Marcy l’Etoile, France; 2 Liphatech, Bonnel, 47480 Pont du Casse, France

**Keywords:** vitamin K epoxide reductase, vitamin K status, mutation, osteocalcin

## Abstract

Vitamin K is crucial for many physiological processes such as coagulation, energy metabolism, and arterial calcification prevention due to its involvement in the activation of several vitamin K-dependent proteins. During this activation, vitamin K is converted into vitamin K epoxide, which must be re-reduced by the VKORC1 enzyme. Various *VKORC1* mutations have been described in humans. While these mutations have been widely associated with anticoagulant resistance, their association with a modification of vitamin K status due to a modification of the enzyme efficiency has never been considered. Using animal models with different *Vkorc1* mutations receiving a standard diet or a menadione-deficient diet, we investigated this association by measuring different markers of the vitamin K status. Each mutation dramatically affected vitamin K recycling efficiency. This decrease in recycling was associated with a significant alteration of the vitamin K status, even when animals were fed a menadione-enriched diet suggesting a loss of vitamin K from the cycle due to the presence of the *Vkorc1* mutation. This change in vitamin K status resulted in clinical modifications in mutated rats only when animals receive a limited vitamin K intake totally consistent with the capacity of each strain to recycle vitamin K.

## 1. Introduction

K vitamins are a complex family composed of several similarly fat-soluble molecules having a 2-methyl-1,4-napthoquinone ring in common. The different forms of vitamin K are distinguished according to the length and nature of the carbon chain in position 3 of the naphthoquinone ring [1]. Vitamin K1 or phylloquinone contains a phytyl side chain, which comprises 4 prenyl units. Vitamin K1 is synthesized only by plants, green algae, and some cyanobacteria and is, thus, found mainly in green vegetables. Vitamins K2 or menaquinones (MK) contain an unsaturated aliphatic side chain with a variable number of prenyl units. Depending on the number of prenyl units, vitamins K2 can be divided into different short-chain MK (i.e., mainly menaquinone-4, MK-4) found mainly in meat and fermented foods and long chain MKs mainly of bacterial origin (i.e., MK-7, to MK-14). 

Vitamin K is important for many physiological processes as the cofactor of the gamma-glutamyl carboxylase (GGCX) enzyme [2,3]. This enzyme is involved in the activation of many proteins, called vitamin K-dependent proteins (VKDPs), by catalyzing their γ-glutamyl carboxylation. Vitamin K is, thus, crucial for blood coagulation through the activation of hepatic blood clotting factors II, VII, IX, and X. Vitamin K is also essential for energy metabolism and arterial calcification prevention through the activation of osteocalcin [4] and Matrix Gla Protein [5], respectively, which are both extrahepatic VKDPs. It was also described to be involved in bone metabolism [6,7], cancer progression, inflammatory response [8], oxidative stress [9], shingolipids synthesis [10], and pancreas exocrine activity [11].

Recommended vitamin K intake in a normal healthy adult varies from 50 to 600 μg/day for vitamin K1, and from 5 to 600 μg/day for vitamin K2 [12]. This recommended vitamin K intake is only based on blood coagulation and varies according to the region. Common polymorphisms or mutations in certain key genes involved in vitamin K metabolism might affect nutritional requirements. Nevertheless, this evidence is indirect via effects on warfarin dose requirements and direct demonstrations are missing.

During gamma-carboxylation of VKDP, vitamin K is converted into vitamin K epoxide by GGCX [2]. The latter is then re-reduced first into vitamin K quinone and then into vitamin K hydroquinone by the vitamin K epoxide reductase (VKORC1) enzyme to be reusable for a new activation of VKDP. It is estimated that one molecule of vitamin K can be used up to 500 times by GGCX [13]. Inhibition of the VKORC1 by vitamin K antagonists such as warfarin leads to inhibition of recycling, which results in a hemorrhage due to a deficiency of activated clotting factors II, VII, IX, and X. This is indicative of the central role played by VKORC1 in the low vitamin K requirement, and any change in this activity may lead to an increased or decreased vitamin K requirement [14].

Various polymorphisms and mutations of the *VKORC1* gene have been described in humans. Five common non-coding polymorphisms of the *VKORC1* gene located in the promoter region, in the first and second intron, and in the 3′ downstream region described in patients requiring increased doses of vitamin K antagonists are associated with reduced expression of VKORC1 [15]. More than 30 mutations in the coding region of *VKORC1* have also been described in patients requiring increased doses of anticoagulants [16,17,18,19,20]. While these mutations have been widely suspected of causing anticoagulant resistance, their association with an increase in the vitamin K requirement due to a modification of the enzyme efficiency has rarely been considered [21].

*Vkorc1* mutations are also widespread in other animal species, particularly rats and mice whose populations are managed with vitamin K antagonists [22]. Recently, characterization of an animal model of rats carrying a *Vkorc1* mutation in the coding region leading to the Y139C mutation has demonstrated that the latter fed with a vitamin K3 deficient diet, which is a synthetic vitamin K normally widely present in rodent diets, presented arterial media calcification [23]. These calcifications were shown to be associated with a gamma-carboxylation defect of Matrix Gla Protein (MGP) involved in the vascular calcification prevention. This gamma-carboxylation defect could be due to a decrease in the efficiency of VKORC1 activity. This work aims to compare the vitamin K status in different rats carrying different mutations of the *Vkorc1* gene in order to evaluate the impact of such mutations and the influence of the nature of the mutations on the vitamin K status and, therefore, on the vitamin K requirement.

## 2. Materials and Methods 

### 2.1. Animals

Rats homozygous for Y139C-*Vkorc1*, rats homozygous for Y139F-*Vkorc1*, and rats homozygous for L120Q-*Vkorc1* were derived from back-crossings and inter-crossings between laboratory Sprague-Dawley rats (designed as recipient strain) (Charles River, St Germain sur l’Arbresles, France) and wild rats homozygous for Y139C-*Vkorc1*, Y139F-*Vkorc1,* or L120Q-*Vkorc1* (designed as a donor strain for the *Vkorc1* mutation). Wild rat strain, carrying the Y139C mutation in *Vkorc1* at the homozygous state, was a generous gift from Julius Kuhn Institute [23]. A wild rat strain carrying the Y139F mutation in *Vkorc1* at the homozygous state was initially trapped on French farms in the 1980s and have since been maintained at the Lyon College of Veterinary Medicine, France [24]. Berkshire rats homozygous for Q120 in the *Vkorc1* were a generous gift from U.K.-based Sorex Ltd., Widnes, Great Britain. The founder animals of this strain were initially trapped in an English farm in the Berkshire countryside [25,26].

To form congenic strains, male wild rats homozygous for Y139C-*Vkorc1*, Y139F-*Vkorc1,* or L120Q-*Vkorc1* were crossed with Sprague-Dawley females to create the F1 hybrid generation, respectively. The F1 heterozygous for the Y139C-*Vkorc1*, Y139F-*Vkorc1,* or L120Q-*Vkorc1* mutation males were backcrossed to Sprague-Dawley females to give the F2 generation. The genotype of F2 young rats was determined by the allele-specific PCR method, as described by References [23,24,26]. Heterozygous females were back-crossed to the recipient Sprague-Dawley strain for 18 additional generations yielding F20 generation. Lastly, an F20 intercross of males heterozygous for Y139C-*Vkorc1*, Y139F-*Vkorc1*, or L120Q-*Vkorc1* with females heterozygous for Y139C-*Vkorc1*, Y139F-*Vkorc1,* or L120Q-*Vkorc1*, respectively, was carried out to obtain F20-introgressed rats homozygous for Y139C-*Vkorc1,* Y139F-*Vkorc1,* or L120Q-*Vkorc1* [23,24,26].

Animals were kept in standard cages (Eurostandard, Type IV, Tecniplast, Limonest, France), and received standard feed (Scientific Animal Food and Engineering, reference A04) and water *ad libitum*. 

### 2.2. Animal Treatment

Experimental research on the rats was performed according to an experimental protocol following international guidelines and with approval from the ethic committee of the Veterinary School of Lyon (authorization n°2017041910166230).

During vitamin K deficiency experiments, eight-week-old male rats received for a maximum of 12 days a diet deficient in vitamin K_3_ (menadione) without barley (Scientific Animal Food and Engineering, SAFE A04 v231) (designed in this study as diet-K3) *ad libitum*. This diet was composed of corn starch 36.1% (instead of barley rich in vitamin K_1_), wheat 25%, corn 15%, wheat bran 5%, peanut oil 3%, soybean meal 8%, fish hydrolyzate 4%, and minerals/vitamins mix 3.9% (without vitamin K_3_). Concentrations of vitamin K_1_ and K_3_ were determined by the supplier and were under the limit of quantification.

Rats were killed 0, 4, 8, or 12 days after the beginning of the feeding period with the special diet -K3 with CO_2_. The organs (liver, kidney, lung, heart, and testis) of each rat were immediately collected and stored at −20 °C.

### 2.3. Preparation of Liver Microsomes

Microsomes were prepared from fresh organs by differential centrifugation, as described by Reference [27]. Livers, lungs, kidneys, and testis of rats fed with a standard diet were resuspended in 50 mM phosphate buffer (pH 7.4) containing 1.15% (*w*/*v*) of KCl. Organ cells were broken and homogenized in buffer by using a motor driven Potter glass homogenizer and further submitted to differential centrifugation at 4 °C. The 100,000 g pellet corresponding to the membrane fraction was resuspended by Potter homogenization in HEPES glycerol buffer (50 mM HEPES, 20% glycerol, pH 7.4). Protein concentrations were evaluated by the method of Bradford [28] using bovine serum albumin as standard microsomes were frozen at −80 °C and used for kinetic analysis and ELISA kits.

### 2.4. Vitamin K Epoxide Reductase Activity (VKOR) Assays and Kinetics

Microsomal vitamin K epoxide reductase (VKOR) activity was assayed as described previously [29]. Standard reactions were performed in 200 mM Hepes buffer (pH 7.4) containing 150 mM KCl, 1 mM dithiothreitol, and 1 g.L-1 of total proteins. The reaction was started by the addition of vit K1 > O solution in 1% Triton X-100 and incubated at 37 °C for 30 min. In these conditions, the reaction was linear, according to the time of incubation and the quantity of incubated proteins. After incubation at 37 °C for 30 min, the reaction was stopped by adding 2 mL of isopropanol. After centrifugation at 3000× *g* for 10 min in order to precipitate proteins, 2 mL of hexane was added. After centrifugation at 3000× *g* for 10 min, the hexane layer was removed and dried under nitrogen. The dry residue was immediately dissolved in 0.2 mL of methanol and the reaction product was analyzed by liquid chromatography-mass spectrometry [29]. 

### 2.5. *VKORC1* and *VKORC1L1* ELISA Quantitative Assays

The VKORC1 protein was quantified from liver, testis, kidney, and lung microsomes using the “Rat Vitamin K epoxide reductase complex subunit 1” ELISA kit (Cliniscience, Nanterre, France), according to the manufacturer’s recommendations. The VKORC1L1 protein was quantified from liver, testis, kidney, and lung microsomes using the “Rat Vitamin K epoxide reductase complex Subunit 1-Like Protein 1” ELISA kit (Cliniscience, Nanterre, France), according to the manufacturer’s recommendations.

### 2.6. Measurement of Vitamin K Concentrations

Furthermore, 0.5 g of tissue was homogenized with ethanol 33% using an Ultra Turrax tissue disperser from IKA Labortechnick^®^ (VWR International, Strasbourg, France and then extracted with 4 mL of hexane. After centrifugation at 3000 rpm for 5 min at 4 °C, the supernatant was collected and extracted again with 4 mL of hexane. After centrifugation at 3000 rpm for 5 min at 4 °C, the supernatant was collected and evaporated at 37 °C to dryness under a gentle stream of nitrogen. The final dry extract was dissolved in 500 µL of methanol and vitamins K (i.e., Menaquinone 4-MK4-, Menaquinone 4 epoxide-epoxide -K1OX-) were analyzed by HPLC on reverse phase C18 column (4.6 × 100 MK4OX-, phylloquinone -K1-, phylloquinone mm, 2.7 µm, VWR, Fontenay-sous-bois, France). A post-column reactor (2.1 × 50 mm steel column packed with zinc powder) was connected in series between the analytical column and detector to ensure the reduction of K vitamins and allow the detection of reduced K vitamins by fluorescence with excitation and emission wavelengths of 246 and 430 nm, respectively. A gradient elution system was used with a flow rate of 1 mL/min as follows. After 24 min of elution with 99% methanol/1% water (acidified with 1% acetic acid containing 1.1 g.L^−1^ of zinc acetate), the mobile phase was quickly changed from 99% methanol/1% water (acidified with 1% acetic acid containing 1.1 g.l^−1^ of zinc acetate) to 50% methanol/49% dichloromethane with 1% acetic acid and 1.1 g.l^−1^ of zinc acetate in 0.1 min. Under these HPLC conditions, MK4OX, MK4, K1OX, and K1 were eluted at 7.5 min, 10.3 min, 12.5 min, and 18.8 min, respectively.

### 2.7. Undercarboxylated Osteocalcin (ucOC) and Undercarboxylated Matrix Gla Protein (ucMGP) ELISA Quantitative Assays

The ucOC concentration was quantified in plasma by ELISA using the “Rat Glu-osteocalcin High Sensitive EIA” kit (Ozyme, Saint Cyr l’école, France), according to the manufacturer’s recommendations.

### 2.8. Tissue Calcium Measurement

Tissues were placed in an oven at 50 °C for 72 h and then reduced to a powder using a glass mortar. Calcium was extracted from 10 to 50 mg of tissue powder (depending on the organs) in 200 µL to 1 mL of a 10% formic acid solution for at least 2 h. The supernatant was recovered after centrifugation at 3000× *g* for 10 min and then evaporated at 110 °C. Residues were resuspended with mili-Q water corresponding to half of the volume of the formic acid solution. Calcium was assayed using the calcium colorimetric assay kit (Sigma-Aldrich, l’Isle d’Abeau, Chesnes, France), according to the manufacturer’s recommendations.

### 2.9. Data Analysis

*K*_m_ and *V*_max_ values were obtained from at least four separate experiments performed on four different batches of protein. The estimation of *K*_m_ and *V*_max_ values was achieved by the incubation of at least nine different concentrations of vit K > O (from 0.003 to 0.2 mM) to the standard reaction. Incubations were performed in duplicate. Data were fitted by nonlinear regression to the Michaelis-Menten model using the R-fit program. 

Data are presented as the mean ± SD. Statistical analysis was done by using the Tukey’s multiple comparisons test or the Dunn’s multiple comparisons tests, and using GraphPad Prism 6 software (GraphPad, San Diego, CA, USA). *p* < 0.05 was the accepted level of significance.

## 3. Results

### 3.1. Tissue VKOR Activity in Rats According to Vkorc1 Genotype

#### 3.1.1. Kinetics Parameters of VKOR Activity

To evaluate the influence of the *Vkorc1* genotype on the ability to recycle vitamin K epoxide in vitamin K quinone between rat tissues, liver, kidney, testis, and lung microsomes from male rats with a different *Vkorc1* genotype (i.e., homozygous for wild type, Y139C, Y139F, or L120Q) were prepared. Catalytic properties of the VKOR activity measured in these microsomes were determined. *Km* and *Vmax* results are presented in Table 1. For all strains, VKOR activity was higher in liver, then in testis, then in kidneys, and then in lungs. Statistical differences were found between strains only concerning the *Vmax* value. The *Vmax* value of VKOR activity in liver of rats belonging to the wild type (WT) strain was, respectively, 4.1, 2.8, and 2.8-fold higher than that of rats belonging to L120Q, Y139F, and Y139C strains. The *Vmax* value of VKOR activity in the testis of rats belonging to the WT strain was, respectively, 6.0, 6.0, and 3.1-fold higher than that of rats belonging to L120Q, Y139F, and Y139C strains. The *Vmax* value of VKOR activity in the kidney of rats belonging to the WT strain was, respectively, 4.2, 4.0, and 2.3-fold higher than that of rats belonging to L120Q, Y139F, and Y139C strains. In lungs, VKOR activity was similar between strains.

#### 3.1.2. Ratio Between VKORC1 and VKORC1L1 Enzymes

Because two different proteins, VKORC1 and VKORC1L1, may be involved in the reduction of vitamin K epoxide in vitamin K quinone, concentrations of both enzymes in liver, testis, kidneys, and lungs were determined by ELISA (Enzyme-linked immunoabsorbent assay) quantitative assays. The results are presented in Table 2. No statistical difference for the ration between VKORC1 and VKORC1L1 was observed between strains. In liver, whatever the strain considered, the concentration of the VKORC1 protein was always much higher than that of the VKORC1L1 protein with no statistical difference between strains except for the Y139F strain for which VKORC1 was 1.5-fold higher than that of the WT strain. In other tissues, concentrations of VKORC1 and VKORC1L1 proteins were comparable with the VKORC1 protein, which is slightly more present than the VKORC1L1 protein in testis and lung. This is the opposite in the kidney. In the lung, VKORC1L1 expression was 1.5 to 1.9-fold greater in mutant strains when compared to the WT strain.

### 3.2. Vitamin K Status According to the Vkorc1 Genotype in Rats fed with a Standard Rodent Diet 

#### 3.2.1. Vitamin K Concentrations

**Figure 1:**
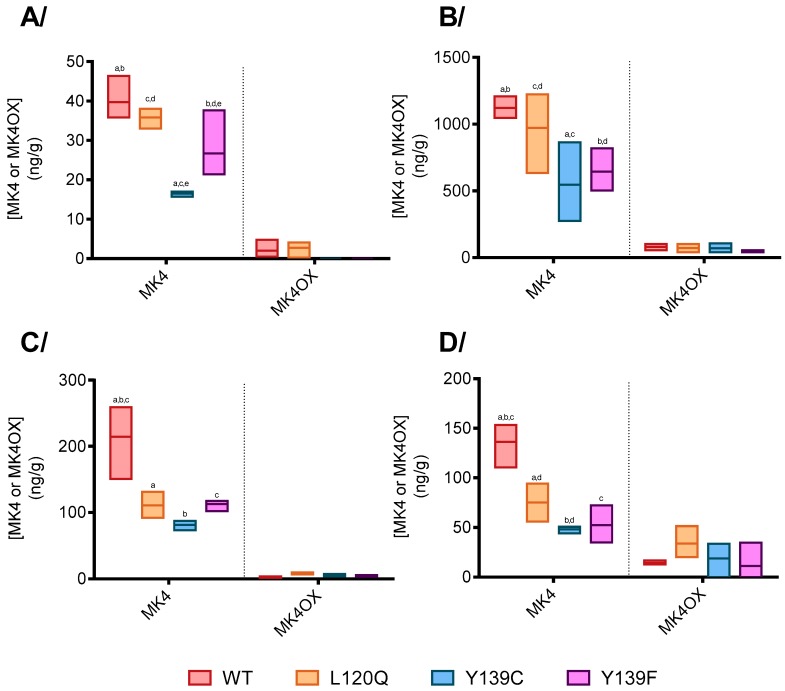
Vitamins K concentrations in (**A**)/liver, (**B**)/testis, (**C**)/kidney, and (**D**)/lung of rats homozygous for WT-, L120Q-, Y139C-, or Y139F-*Vkorc1* fed with a standard rodent diet. The box extends from the 25th to 75th percentiles and the line corresponds to the mean. Statistical analyses were performed using a Tukey’s multiple comparisons test. *p* < 0.05 was the accepted level of significance. a,b,c,d,e, statistical difference between 2 groups.

To evaluate the influence of the *Vkorc1* genotype on the vitamin K status, concentrations of vitamin K1, K1OX, MK4, and MK4OX were determined in liver, testis, kidneys, and lungs of rats homozygous for wild type, Y139C, Y139F, or L120Q fed with a standard rodent diet. Vitamin K1 and K1OX were almost undetectable in all the considered tissues, whatever the genotype of rats. Only MK4 and MK4OX were, therefore, quantified. Results are presented in Figure 1. Statistic differences were found between strains only concerning the concentration of MK4.

Whatever the tissues considered, MK4 concentration was systematically higher in rats homozygous for wild type *Vkorc1* compared with rats homozygous for Y139F-*KORC1* or Y139C- *KORC1*. In liver, it was 1.5-fold and 2.4-fold higher. In testis, it was 1.7-fold and 2.1-fold higher and, in kidneys, it was 1.9-fold and 2.6-fold higher. In the lung, it was 2.7-fold and 2.8-fold higher when compared with rats homozygous for Y139F-*Vkorc1* or Y139C-*Vkorc1*, respectively.

In liver and testis, MK4 concentration was similar between wild type rats and rats homozygous for L120Q, while it was 1.9-fold higher in lungs and kidneys of wild type rats compared with rats homozygous for L120Q.

#### 3.2.2. Plasma ucOC Concentration 

**Figure 2:**
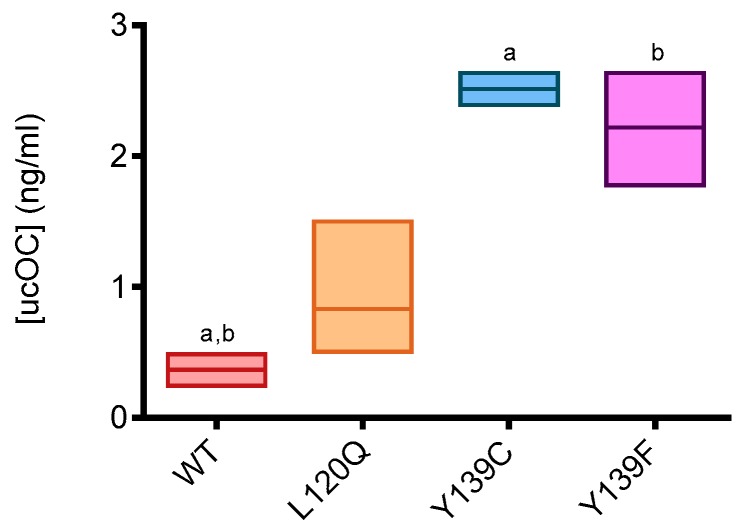
ucOC concentrations in the plasma of rats homozygous for WT-, L120Q-, Y139C-, or Y139F-*Vkorc1* fed with a standard rodent diet. The box extends from the 25th to 75th percentiles and the line corresponds to the mean. Statistical analyses were performed using a Dunn’s multiple comparisons test. *p* < 0.05 was the accepted level of significance. a,b, statistical difference between two groups.

Under-gamma carboxylated osteocalcin (ucOC) and matrix Gla protein (ucMGP) concentration was measured in plasma of rats homozygous for wild type, Y139C, Y139F, or L120Q fed with a standard rodent diet. Results for ucOC are presented in Figure 2. Statistical differences were found only for ucOC concentration between wild type rats and rats homozygous for Y139C and Y139F. UcOC concentration was 6.1-fold and 7.0-fold higher in the plasma of rats homozygous for Y139F- and Y139C-*Vkorc1*, respectively, compared with wild type rats. A difference between wild type rats and rats homozygous for L120Q was not statistically significant.

#### 3.2.3. Tissue Calcium Concentration

**Figure 3:**
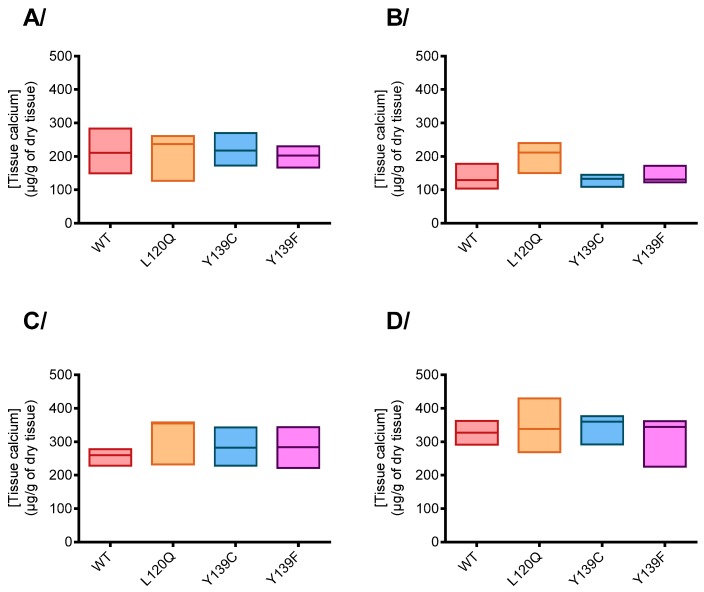
Tissue calcium content in (**A**) testis, (**B**) kidney, (**C**) lung, and (**D**) heart of rats homozygous for WT-, L120Q, Y139C or Y139F-*VKORC1* fed with a standard rodent diet. The box extends from the 25th to 75th percentiles and the line corresponds to the mean. Statistical analyses were performed using a Dunn’s multiple comparisons test.

Calcium concentration was measured in testis, kidneys, lungs, and hearts of rats homozygous for wild type, Y139C, Y139F, or L120Q fed with a standard rodent diet. Results are presented in Figure 3. No difference was observed between strains.

### 3.3. Evolution of the Vitamin K Status According to the Vkorc1 Genotype in Rats Receiving a Specific Diet-K3 for 12 Days

#### 3.3.1. Vitamin K Concentrations

**Figure 4:**
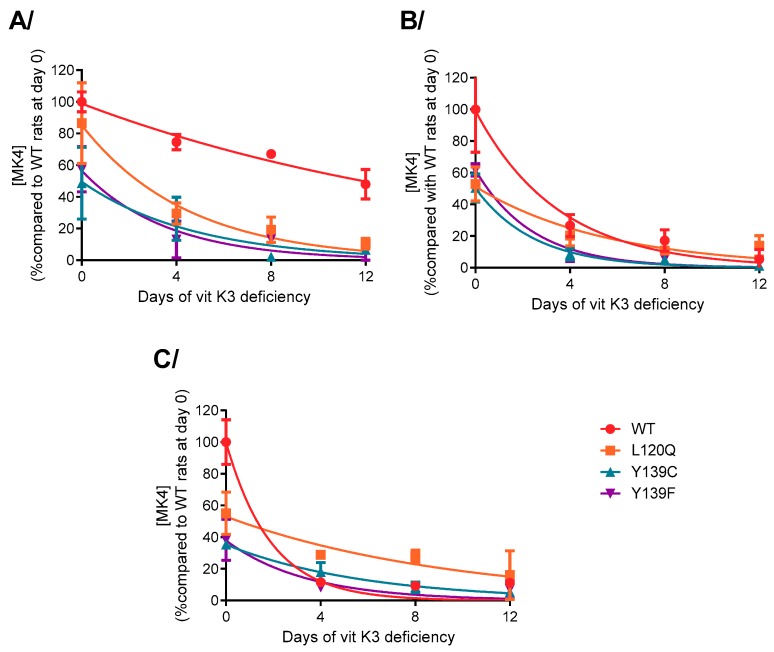
Vitamins K concentrations evolution in (**A**) testis, (**B**) kidney, and (**C**) lung of rats homozygous for WT-, L120Q-, Y139C-, or Y139F-*Vkorc1* receiving a specific diet -K3 for 12 days. Results are expressed as the mean of the percentage of four animals compared to wild type rats at day 0 ± standard deviation.

The evolution of MK4 concentrations in different tissues (i.e., testis, kidneys, and lungs) was determined in rats receiving a vitamin K3 deficient diet for a short period of time from 0 to 12 days. The results are presented in Figure 4. This evolution could not be determined in the liver since this concentration was too low even before the beginning of the deficiency.

Half-lives of MK4 in the different tissues of the different rat strains were calculated using a non-linear regression model using equation C = (C0)^-kel*t^. Results are presented in Table 3. Half-life of MK4 was four-fold longer in testis of the wild type when compared to mutated rats. In kidney, half-lives were similar for all strains. In lung, half-life of MK4 was lower in wild type rats when compared to rats homozygous for L120Q or Y139F.

#### 3.3.2. Prothrombin Time

**Figure 5:**
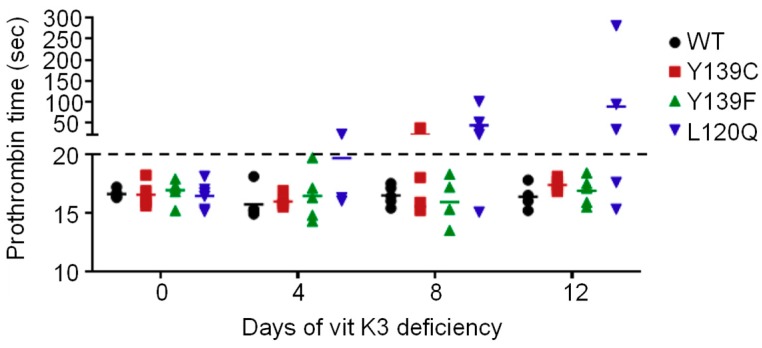
Prothrombin time evolution of rats homozygous for WT-, L120Q-, Y139C-, or Y139F-*Vkorc1* receiving a specific diet -K3 for 12 days. Results are the mean of 3 or 5 animals, with each point representing each individual.

Consequences of the vitamin K3 deficiency were assessed by the determination of the prothrombin time at day 0, 4, 8, and 12 after the beginning of the vitamin K3 deficiency. Results are presented in Figure 5. Prothrombin time was not modified in rats homozygous for the wild type, Y139C, or Y139F fed with a standard rodent diet. Prothrombin time increased in rats homozygous for L120Q from the eighth day after the beginning of the vitamin K3 deficiency.

#### 3.3.3. Plasma ucOC Concentration

**Figure 6:**
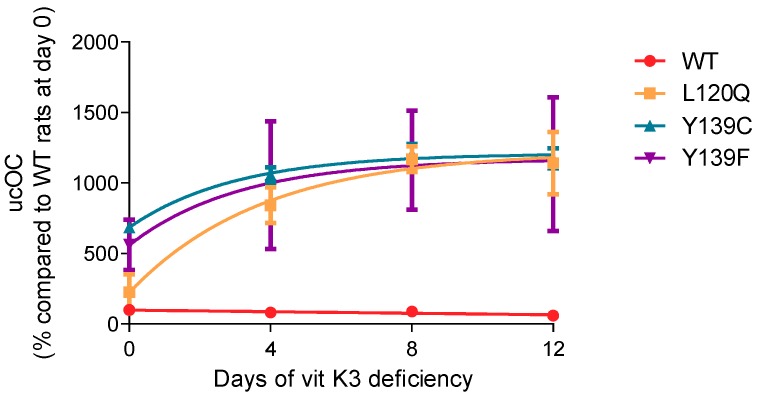
ucOC concentrations evolution in plasma rats homozygous for WT-, L120Q-, Y139C-, or Y139F-*Vkorc1* receiving a specific diet -K3 for 12 days. Results are expressed as the mean of the percentage of 4 animals compared to wild type rats at day 0 ± standard deviation.

Consequences of the vitamin K3 deficiency were also assessed by determining the plasma ucOC concentration at day 0, 4, 8, and 12 after the beginning of the vitamin K3 deficiency. Results are presented in Figure 6. The ucOC concentration remained unchanged during the 12 days of vitamin K3 deficiency in wild type rats, while, in the other strains, ucOC concentration increased rapidly. ucOC concentration reached its maximum 4 days after the beginning of the vitamin K3 deficiency and increased two-fold in rats homozygous for Y139F and Y139C and four-fold in rats homozygous for L120Q.

## 4. Discussion

Anticoagulant-resistant rats due to *Vkorc1* gene mutations are good models for mimicking the consequences of human *VKORC1* mutations. In humans, mutations of the *VKORC1* promoter would decrease VKORC1 expression and some VKORC1 coding mutations would affect its VKOR activity [30]. The consequences of these mutations may, therefore, be different, in terms of activity and/or the consequences on vitamin K status. Using the rat as a model, we have considered these possible differences depending on the nature of the mutation. For this, we used four different rat strains, the OFA-Sprague Dawley rats, and three strains of introgressed rats homozygous for mutations L120Q, Y139F, or Y139C, with the amino acid 139 being located near the catalytic site, and the amino acid 120 being further away from the latter [31]. These strains were derived from the OFA-Sprague Dawley rat in which the mutated *Vkorc1* gene was introduced with less than 0.0001% of the initial genome of the wild brown rat. We can, thus, legitimately affect the phenotypic modifications to the *Vkorc1* mutation.

We first assessed the impact of the mutation on the ability of each tissue to recycle vitamin K. For this, we measured VKOR activity from liver, kidney, lung, and testicular microsomes. VKORC1 is localized in the membrane of the endoplasmic reticulum [2]. The liver is responsible for the activation of factors II, VII, IX, and X {2]. The kidney, lung, and testis contain high amounts of vitamin K [32] and calcify strongly in the absence of vitamin K [23]. Regardless of the tissue, microsomal VKOR activity is always greatly decreased when VKORC1 is mutated, regardless of the position (i.e., amino acid 120 or amino acid 139) and nature (whether amino acid 139 is replaced by phenylalalanine or cysteine) of the mutation. Affinity for vitamin K does not seem to be modified (*Km* unchanged), but velocity of VKOR activity is systematically greatly reduced (by a factor of 3 to 6 depending on the tissue and the mutation). This loss of velocity may be due to a malfunction of VKORC1 due to mutations and/or a decrease in the VKORC1 expression level because of instability of the mutated proteins. The malfunction is certain. The characterization of the corresponding recombinant proteins has demonstrated this loss of velocity [33]. On the other hand, this study shows that the VKORC1 expression level remains unchanged despite the presence of mutations L120Q, Y139F, or Y139C regardless of the tissue, except in the liver of the Y139F strain where VKORC1 is slightly expressed. Expression of VKORC1L1, which is another enzyme that is able to catalyze VKOR activity [34] and, thus, compensate for loss of VKORC1 activity, also remains unchanged except in lungs where its expression is slightly increased to compensate for the loss of activity of VKORC1 in the lung.

The presence of these mutations in rats that can be considered laboratory rats allowed us to appreciate the consequences of a decrease in VKOR activity on the “vitamin K” status (Table 4). This status was evaluated by determining tissue vitamin K concentrations, measurement of plasma ucOC concentration, and determination of tissue calcium levels. Among the K vitamins, only MK4 was measured because the rats used received, from the age of 3 weeks up to 8 weeks, a diet supplemented with menadione as their sole source of vitamin K (the amounts of vitamin K1 in the standard feed being limited and provided by the cereals incorporated). The latter received virtually no vitamin K1 and, since there is no endogenous synthesis of vitamin K1, vitamin K1 was almost undetectable in rat tissues. Osteocalcin is a vitamin K-dependent protein produced mostly by osteoblasts [35]. In the absence of vitamin K, the concentration of ucOC increases rapidly in plasma [36] and is, therefore, considered a good marker of overall vitamin K status. Tissue calcium concentrations have also been shown to be negatively correlated with the vitamin K status. In the absence of vitamin K, MGP, which is another vitamin K-dependent protein, cannot be activated and, therefore, cannot inhibit tissue calcification [23,37,38].

Surprisingly, while all rats have always received the same standard diet supplemented with menadione, the vitamin K status of rats homozygous for Y139C and Y139F mutations is strongly altered with a sharp decrease (by a factor of two to four) in MK4 concentrations in all tissues considered in this study and a significant increase in plasma ucOC concentration (by a factor of almost 7). The “vitamin K” status of rats homozygous for the L120Q mutation can be considered an intermediate between the wild type rat and the rats homozygous for the Y139C or Y139F mutations (Table 4). It is surprising to observe such a difference in vitamin K status in rats receiving the same diet supplemented with menadione, even if they have *Vkorc1* mutations. This decrease is even more surprising in the liver, which is the first organ to capture vitamin K from food, where the intake is the same for all strains of rats. Decreasing the recycling efficiency of vitamin K should eventually lead to a decrease in MK4 concentration, but an increase in MK4OX concentration. This is not observed. These results suggest a loss of vitamin K induced by the mutation. The metabolism of vitamin K is still largely unknown. Metabolism of vitamin K by ω-hydroxylation [39,40] with involvement of cytochromes 4F2 and 4F11 in humans has been described. The substrates of cytochromes 4F2 and 4F11 have not been characterized. In rats, cytochromes metabolizing vitamin K have not been identified. Metabolism of vitamin K in its epoxide form by cytochromes might be more efficient than in its quinone form. Since recycling of vitamin K epoxide is less efficient in mutated rats, the elimination of vitamin K could be, thus, increased via greater cytochrome P450 dependent metabolism of vitamin K epoxide. Nevertheless, the difference in status observed between rats homozygous for the L120Q mutation and rats homozygous for the Y139F and Y139C mutations is surprising, whereas the recycling efficiency of the different tissues is similar, or even lower, in rats homozygous for the L120Q mutation (the efficiency that corresponds to *Vmax*/*Km* is systematically the lowest). The “vitamin K” status of rats homozygous for the Y139F or Y139C mutations could be aggravated by a modification of the catalysis mediated by VKORC1 due to the mutation. It has been shown that the replacement of tyrosine 139 by cysteine, and, to a lesser extent by phenylalanine, allowed VKORC1 to catalyze a 3-hydroxylation activity, in addition to its activity of reduction [33,41]. The production of 3-hydroxyvitamin K constitutes an additional pathway of elimination of vitamin K and, thus, precludes the recycling of the vitamin by increasing the vitamin K requirements in these rats. Wild type VKORC1 and VKORC1 mutated at position 120 do not produce this 3-hydroxyvitamin K [33].

The modification of the vitamin K status in the mutated rat strains does not appear to have apparent clinical consequences when animals receive a diet supplemented with menadione. In fact, the rats do not show any changes in coagulation (the quick times are comparable between strains) and do not seem to have calcification problems (tissue calcium concentrations are also comparable between strains). On the other hand, it has been shown that rats homozygous for the Y139C mutation receiving a synthetic diet (cereals replaced by starch) not supplemented with menadione for three months presented very strong vascular calcifications in testes, lungs, and kidneys [23]. This is why we evaluated the ability of different rat strains to resist a limited vitamin K intake by feeding them a synthetic diet without menadione (diet-K3) for a period of 12 days. To evaluate their resistance, we determined tissue MK4 concentrations (except those in liver because of the extra low concentrations), plasma ucOC concentrations, and prothrombin time, throughout the period of limited intake of vitamin K. Menadione deficiency results very rapidly into lower tissue levels of MK4 in all tissues, regardless of strains. After only four days of menadione deficiency, tissue MK4 concentrations are no longer different between strains (except in testis of wild type rats). In the absence of menadione intake, synthesis of MK4 by the enzyme UBIA1 is stopped [42,43] and tissue MK4 levels decrease to reach the same concentration in all rats. The residual quantities are potentially synthesized by the intestinal microflora [44], with the animals being all housed under the same breeding conditions, in the same rooms, and receiving all the same food. It was discovered that the presence of MK4 in the testis of wild type rats persisted longer. This persistence remains unexplained. The rapid decrease in testis of mutated strains might be due to the needs of vitamin K in other tissues to maintain the pool of activated VKDP.

This rapid decrease in MK4 concentrations is associated with a rapid increase in ucOC concentration in all mutated rats. This increase is even more rapid in the homozygous rats for the L120Q mutation with a concentration of ucOC becoming similar to that of rats homozygous for mutations Y139C and Y139F from the fourth day after the beginning of the deficiency, whereas this concentration was initially lower than that of rats homozygous for mutations Y139C and Y139F. The ucOC concentration remains unchanged in wild type rats. The same findings had already been reported for MGP in the Y139C rat with an increase in tissue ucMGP concentrations [23]. These evolutions are completely consistent with the capacity of each strain to recycle vitamin K. Since dietary vitamin K intake is limited, the gamma-carboxylation of vitamin K dependent proteins is dependent on the recycling of residual concentrations of vitamin K. The wild type rat has optimal recycling and is virtually independent of vitamin K dietary intakes. Mutated rats with limited recycling have high dietary vitamin K requirements and, when vitamin K intake is reduced, activation of vitamin K-dependent proteins becomes insufficient due to a lack of vitamin K quinone. Nevertheless, activation of hepatic vitamin K-dependent proteins (i.e., coagulation factor II, VII IX, and X) appears to be relatively preserved when compared to those of extrahepatic vitamin K dependent proteins (i.e., OC). The prothrombin times remain unchanged throughout the 12 days of deficiency, (with the exception of those of rats homozygous for the L120Q mutation which increase after 8 days of deficiency), whereas the ucOC concentration increases from the fourth day of deficiency. Since the liver is the first organ to capture dietary vitamin K, it is possible that vitamin K1 present in reduced amounts in food may maintain activated clotting factor concentrations at levels consistent with a normal prothrombin time [45]. In rats homozygous for the L120Q mutation, the recycling efficiency being the lowest (3 and 1.5 times lower than the homozygous rats for the Y139F and Y139C mutation, respectively), the low dietary intake is no longer sufficient to maintain the pools of activated clotting factors at sufficient levels.

In this study, we clearly showed that coding mutations of the *Vkorc1* gene could affect the vitamin K status due to failing vitamin K recycling. This change in the vitamin K status has no apparent clinical consequences if vitamin K dietary intake is sufficient. In case of insufficient dietary vitamin K intake, these mutations can lead to insufficient activation of extrahepatic vitamin K-dependent proteins that can lead to vascular calcification due to the absence of MGP activation [23,46] changes in glucose homeostasis due to the increase in plasma ucOC described as an osteoblast-derived hormone [47], or even coagulation problems in the case of mutations severely affecting the efficiency of vitamin K recycling. Nevertheless, during this study, only three mutations were characterized. The three mutated amino acids are all localized in the fourth transmembrane domain containing the catalytic site [31], whereas, in humans, mutations have been detected in the four transmembrane domains and in the cytosolic loop and do not always seem to affect VKORC1 activity [30]. Further studies will be needed to evaluate the impact of each mutation on the vitamin K status. Nevertheless, the prevalence of these mutations in patients with vascular calcifications could be informative with regard to their impact on vitamin K status.

## Figures and Tables

**Table 1:** Kinetics parameters of VKOR (Vitamin K epoxide reductase) activity and ratio between VKORC1 and VKORC1L1 proteins in different rat tissues according to the *Vkorc1* genotype. Results are the mean of four independent determinations with its 95% confidence interval.

Tissue	Strain	*Km* (µM)	*Vmax* (pmol.min^−1^.mg^−1^)	*Vmax*/*Km* (µL.min^−1^.mg^−1^)
**Liver**	WT	29.58 [18.96–40.21]	95.62 ^a,b,c^ [84.13–107.1]	3.2 ^a,b,c^ [2.2–4.2]
	L120Q	37.98 [20.45–55.52]	22.18 ^a,d,e^ [18.45–25.91]	0.6 ^a^ [0.4–0.8]
	Y139F	22.52 ^a^ [14.49–30.56]	34.50 ^b,e^ [30.62–38.39]	1.6 ^b^ [1.3–1.9]
**Testis**	Y139C	46.89 ^a^ [30.64–53.13]	34.52 ^c,d^ [29.97–39.07]	0.8 ^c^ [0.5–1.1]
	WT	9.92 [4.00–15.84]	53.26 ^a,b,c^ [45.03–61.49]	5.5 ^a,b,c^ [5.3–5.7]
	L120Q	16.70 [11.18–22.22]	8.94 ^a,d^ [8.08–9.79]	0.5 ^a^ [0.4–0.6]
	Y139F	5.43 [2.31–8.55]	8.86 ^b,e^ [6.53–11.19]	1.2 ^b^ [0.6–1.8]
	Y139C	12.35 [3.55–21.15]	17.05 ^c,d,e^ [12.11–21.99]	1.4 ^c^ [1.0–1.8]
**Kidney**	WT	12.12 [3.79–20.45]	20.39 ^a,b,c^ [15.42–25.36]	1.7 ^a,b^ [1.0–2.4]
	L120Q	23.32 [19.80–26.84]	4.82 ^a,d^ [4.59–5.05]	0.2^a^ [0.1–0.3]
	Y139F	12.25 [1.48–23.03]	5.11 ^b,e^ [3.89–6.32]	0.5 [0.1–0.9]
	Y139C	23.24 [4.98–41.5]	8.67 ^c,d,e^ [6.06–11.28]	0.4^b^ [0.2–0.6]
**Lung**	WT	13.16 [6.017–20.29]	6.53 ^a,b^ [5.56–7.51]	0.5 [0.2–0.8]
	L120Q	19.24 [14.84–23.63]	3.94 ^a,c^ [3.67–4.21]	0.20 [0.1–0.3]
	Y139F	10.03 [3.4–16.66]	4.11 ^b^ [2.87–5.35]	0.4 [0.2–0.6]
	Y139C	15.78 [3.06–28.50]	6.43 ^c^ [4.59–8.26]	0.4 [0.2–0.6]

*p* < 0.05 was the accepted level of significance. a, b, c, d, e statistical difference between the two groups. WT: wild type.

**Table 2:** VKORC1 and VKORC1L1 concentrations and ratio between VKORC1 and VKORC1L1 proteins in different rat tissues according to the *Vkorc1* genotype. Results are the mean of 4 independent determinations with its standard deviation.

Tissue	Strain	VKORC1 (µg/g of Tissue)	VKORC1L1 (µg/g of Tissue)	[VKORC1 Protein]
				[VKORC1L1 Protein]
**Liver**	WT	4.44 ± 0.97	0.27 ± 0.04	16.2 ± 1.7
	L120Q	2.66 ± 0.38	0.19 ± 0.03	14.0 ± 1.4
	Y139F	7.13 ± 2.30 *	0.43 ± 0.13	17.6 ± 5.7
	Y139C	4.31 ± 0.80	0.30 ± 0.05	14.4 ± 2.2
**Testis**	WT	0.25 ± 0.07	0.19 ± 0.04	1.4 ± 0.5
	L120Q	0.35 ± 0.08	0.21 ± 0.09	1.6 ± 0.5
	Y139F	0.24 ± 0.10	0.22 ± 0.02	1.1 ± 0.5
	Y139C	0.27 ± 0.06	0.21 ± 0.04	1.3 ± 0.3
**Kidney**	WT	0.10 ± 0.03	0.18 ± 0.02	0.54 ± 0.14
	L120Q	0.14 ± 0.02	0.17 ± 0.01	0.82 ± 0.13
	Y139F	0.08 ± 0.04	0.23 ± 0.02	0.33 ± 0.19
	Y139C	0.12 ± 0.01	0.18 ± 0.01	0.67 ± 0.06
**Lung**	WT	1.25 ± 0.12	0.70 ± 0.06	1.8 ± 0.2
	L120Q	1.25 ± 0.22	1.35 ± 0.22 *	0.9 ± 0.2
	Y139F	1.83 ± 0.54	1.22 ± 0.11 *	1.5 ± 0.2
	Y139C	1.23 ± 0.19	1.10 ± 0.04 *	1.1 ± 0.2

* *p* < 0.05 was the accepted level of significance compared to the WT strain.

**Table 3:** Kinetic parameters of VKOR activity and the ratio between VKORC1 and VKORC1L1 proteins in different rat tissues according to the *Vkorc1* genotype. Results are the mean of four independent determinations with its 95% confidence interval.

Half-Life of MK4	WT (in Days)	L120Q (in Days)	Y139F (in Days)	Y139C (in Days)
In testis	12.09 ^a,b,c^ [10.03–13.82]	3.10 ^a^ [2.22–5.11]	3.31 ^b^ [2.07–8.21]	2.50 ^c^ [1.69–4.74]
In kidney	2.43 [1.78–3.84]	3.85 [2.79–6.27]	1.71 [1.31–2.47]	1.68 [1.34–2.28]
In lung	1.31 ^a,b^ [0.91–2.39]	6.60 ^a^ [4.41–13.20]	4.08 ^b^ [3.37–5.18]	2.37 [1.37–8.93]

*p* < 0.05 was the accepted level of significance. a, b, and c: statistical difference between two groups.

**Table 4:** Summary of the results obtained for each strain compared to the WT strain; 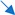, decrease compared to WT strain; 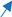, increase for WT strain; **=**, similar with WT strain.

	L120Q	Y139F	Y139C
**VKOR ACTIVITY**			
In liver	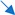 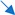	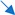	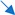
In testis	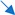 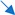	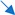 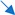	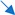
In kidney	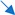 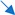	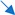 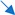	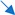
In lung	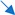	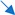	=
**VKORC1 CONCENTRATION**			
In liver	=	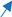	=
In testis	=	=	=
In kidney	=	=	=
In lung	=	=	=
**VKORC1L1 CONCENTRATION**			
In liver	=	=	=
In testis	=	=	=
In kidney	=	=	=
In lung	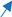	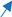	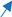
**MK4 CONCENTRATION**			
In liver	=	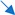	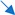 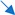
In testis	=	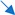	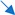 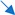
In kidney	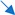 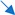	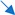 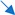	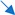 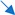
In lung	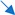	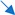	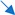 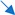
**PLASMA ucOC CONCENTRATION**	**=to** 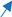	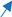	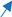
**TISSUE CALCIUM CONCENTRATION**			
In liver	=	=	=
In testis	=	=	=
In kidney	=	=	=
In lung	=	=	=
